# Pneumatosis Intestinalis Manifesting as an Atypical Presentation of Crohn’s Disease

**DOI:** 10.7759/cureus.53151

**Published:** 2024-01-29

**Authors:** Charles Vallejo, Yousra Gheit, Talwinder K Nagi, Zoilo K Suarez, Muhammad Haider, Touqir Zahra

**Affiliations:** 1 Internal Medicine, Florida Atlantic University, Boca Raton, USA; 2 Internal Medicine, Florida Atlantic University Charles E. Schmidt College of Medicine, Boca Raton, USA

**Keywords:** double-balloon enteroscopy, infliximab, ct enteroscopy, crohn’s disease (cd), pneumatosis intestinalis

## Abstract

Crohn’s disease is an inflammatory bowel disease that has a bimodal distribution, occurring most frequently between ages 15 to 30 years and 40 to 60 years. It presents with a relapsing and remitting course. The most common area involved is the terminal ileum and right colon and the inflammation oftentimes leads to non-caseating granulomas and ulcerations in both the superficial mucosa and deeper layers. Additionally, pneumatosis intestinalis is defined as the presence of gas and free air in the extraluminal space of the intestines which is an abnormal occurrence and correlates with underlying pathology. There are only a few cases reported in the literature that present pneumatosis intestinalis in the setting of, and possibly linked to, Crohn’s disease. Our case presents an elderly male patient with jejunal ulcerations and strictures suggesting Crohn’s disease and associated pneumatosis intestinalis as evidenced on outpatient computed tomography (CT) enterography. Upon presentation to the hospital, the patient was non-toxic and was not complaining of any pain. During his inpatient stay, there was a suspicion of Crohn’s disease and therefore he was started on Infliximab therapy. We will review the possible pathogenesis of Crohn’s disease and other cases presenting pneumatosis intestinalis in the setting of Crohn’s disease.

## Introduction

Crohn’s disease is a relapsing and remitting inflammatory bowel disease that may extend from the mucosa to the serosa, otherwise known as the entirety of the bowel wall. The exact etiology is unknown and the pathophysiology is multifactorial, but it is thought that factors such as drugs, toxins, infections, or intestinal microbes may play an important role in a genetically susceptible host [1]. Although the terminal ileum is the most commonly involved site, Crohn’s disease may actually affect any part of the gastrointestinal tract, ranging from the mouth to the perianal area. Histology of the affected parts may reveal non-caseating granulomas, while gross appearance depicts the classic “cobblestone” pattern, which refers to alternating diseases and normal, spared areas. There are quite a few complications associated with Crohn’s disease including chronic inflammation that leads to scarring with subsequent strictures and obstruction [1].

Treatment and management may be broadly grouped into two classes. If the disease is mild to moderate, patients can be treated with steroids, methotrexate, or immunomodulators [1]. If the disease is moderate or severe, patients may be treated with a combination of immunomodulators and biologics or biologics alone [1]. Some common biologics include infliximab and adalimumab. Surgical treatments are usually reserved for complications related to Crohn’s disease such as bowel obstructions, abscess, fistulas or perforated bowel. In general, there is no cure for Crohn’s disease and most patients have a reduced life expectancy due to malignancies, genitourinary disease, and liver and biliary tract complications [1].

Moreover, pneumatosis intestinalis (PI) is defined as the presence of gas and free air in the extraluminal space of the intestines which is an abnormal occurrence and correlates with underlying pathology [2]. The prevalence of PI in patients with Crohn's disease has been found to be as high as 12% compared to 0.03% in the general population. However, there are only a few cases reported in the literature that present PI in the setting of, and possibly linked to Crohn’s disease. Our case presents an elderly male patient with jejunal ulcerations and strictures suggesting Crohn’s disease and associated pneumatosis intestinalis as evidenced on outpatient computed tomography (CT) enterography.

Although PI can present asymptomatically in patients with Crohn's disease, it can also manifest in a variety of ways. Specifically, some patients with Crohn's disease and PI have reported symptoms including diarrhea, hematochezia, abdominal pain, tenesmus, and constipation [3].

## Case presentation

The patient is an eighty-five-year-old male with a past medical history significant for prostate cancer status post radical prostatectomy, hypertension, and diverticulosis who presented due to pneumatosis intestinalis of the small bowel, which was seen on an outpatient computed tomography (CT) enterography. The patient had been experiencing unexplained weight loss for the past 18 months and had been undergoing evaluations to determine the etiology of this weight loss. He endorsed complaints of abdominal discomfort due to gas, but denied any other associated gastrointestinal symptoms. Endoscopy and colonoscopy performed six months prior to his presentation showed non-specific inflammation with no evidence of malignancy noted. Then, he had a capsule endoscopy performed in which subsequently, the capsule was found “stuck” in the jejunum, with adjacent associated ulcerations. In order to retrieve the capsule, a double-balloon enteroscopy was utilized and simultaneously found a jejunal stricture along with adjacent ulcerations where the capsule was lodged and extracted. The patient had further work-up of inflammatory markers completed. These markers were slightly elevated with no signs of infection noted. Specifically, C-reactive protein (CRP) was 27 milligrams per liter (mg/l), erythrocyte sedimentation rate (ESR) was 52 millimeters per hour (mm/h), and fecal calprotectin was 142 micrograms per gram (ug/g).

**Figure 1:**
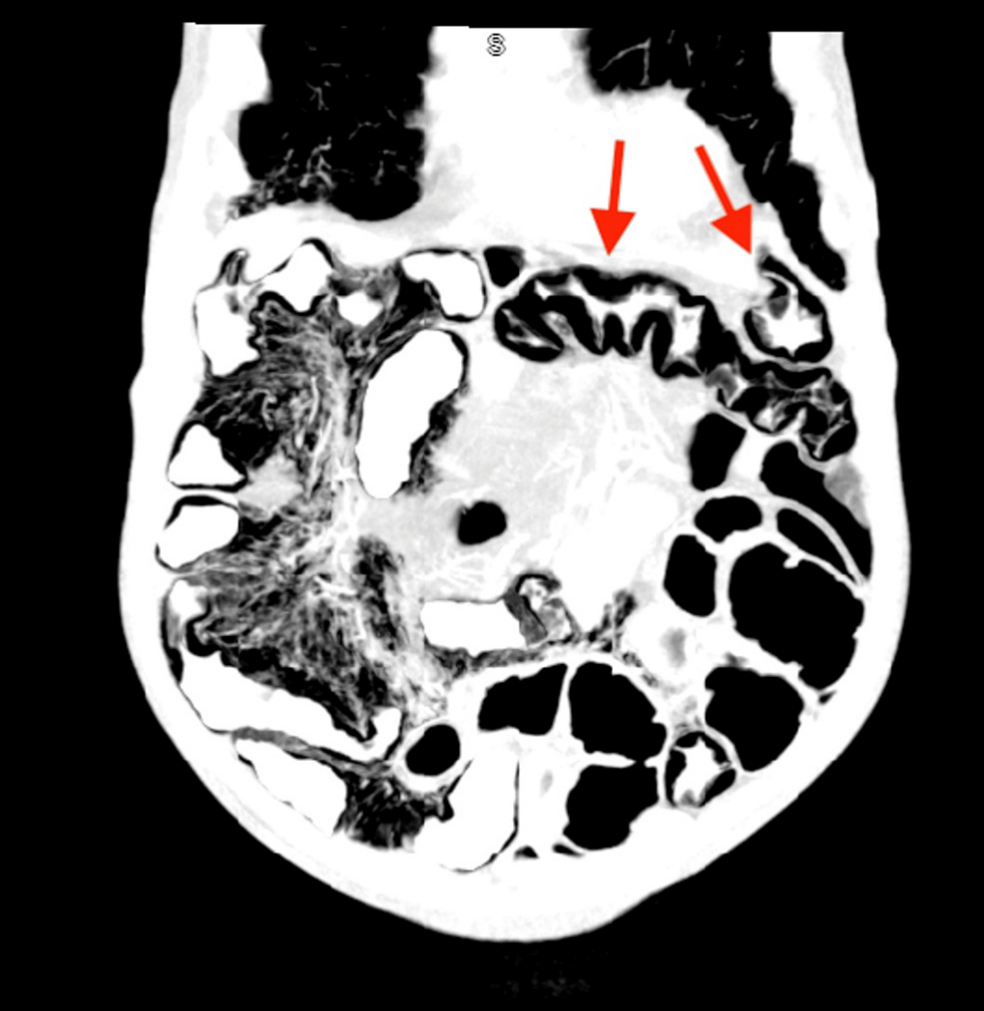
Computed tomography (CT) of the abdomen and pelvis depicting arrows pointing to pneumatosis intestinalis in the jejunum.

**Figure 2:**
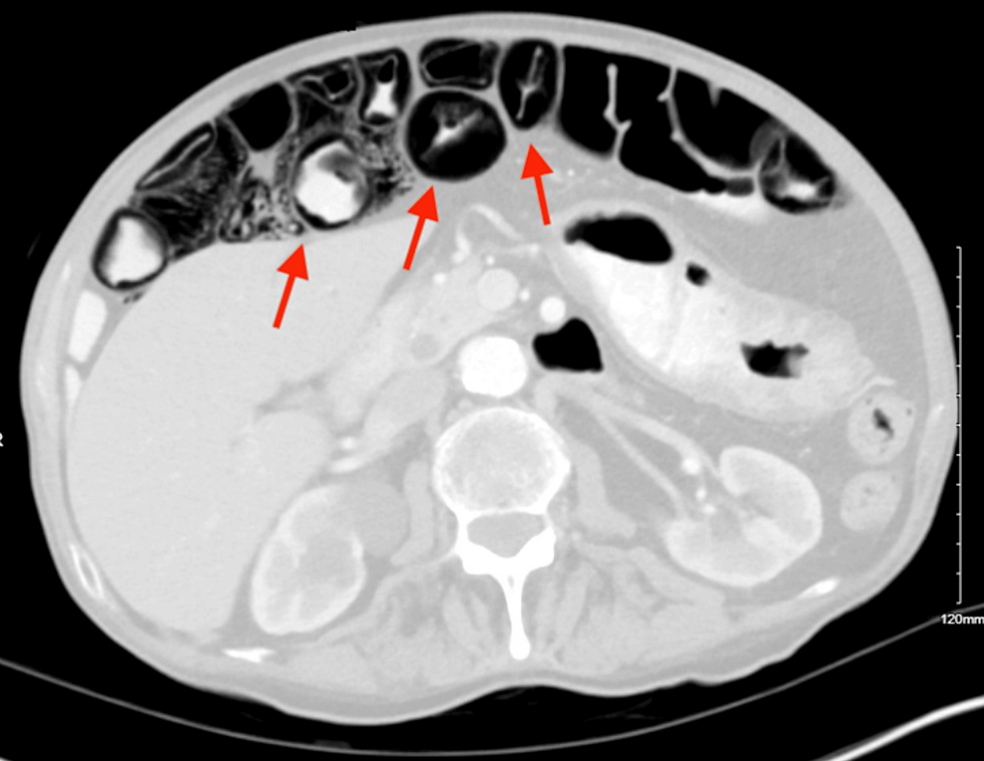
Prominent pneumatosis intestinalis depicted by arrows on the axial view of abdomen and pelvis computed tomography (CT).

Afterwards, the patient was started on prednisone empirically as an outpatient. He then had another CT enterography where pneumatosis intestinalis of the jejunum was noted again. As a result, the patient was recommended to come to the emergency department for admission and further workup on the potential diagnosis of inflammatory bowel disease (IBD) due to these findings. Upon presentation to the hospital, the patient was non-toxic and was not complaining of any pain. He was admitted for further inpatient workup as a concern of pneumatosis intestinalis on outpatient CT enterography imaging. During his inpatient stay, the CT abdomen and pelvis revealed pneumatosis intestinalis in the jejunum as pointed to by the arrows in Figure 1 and Figure 2. Additionally, there was a suspicion of Crohn’s disease and therefore he was started on Infliximab therapy. He was discharged with outpatient gastroenterology follow-up on the second day of admission.

## Discussion

There have been only a few cases of pneumatosis intestinalis occurring in the setting of presumed Crohn’s disease such as the case we present. One case report presents a 60-year-old female patient with long-standing fistulizing Crohn’s disease. During the course of her disease, she required 2 ileocolic resections, of which, one of them was complicated by anastomotic dehiscence. Later, when she presented with her usual mild abdominal discomfort, a follow-up CT scan included cystic pneumatosis intestinalis in the ascending and transverse colon [4]. Another case reported a 58-year-old female patient with long-standing Crohn’s disease complicated by strictures requiring two ileal resection and treatment with sulfasalazine and azathioprine. Three years later, an abdominal CT scan was requested because of mechanical low back pain in which cystic pneumatosis intestinalis was observed in the ascending colon. As a result, gastroenterology was consulted for an emergency case. However, similar to our case, the patient was asymptomatic and laboratory results were insignificant, including no leukocytosis and C-reactive protein of 1.2 milligram/liter (mg/l). The patient was treated conservatively with clinical and radiological monitoring. The authors of these two cases propose that pneumatosis intestinalis has historically been considered a sign of severity associated with intestinal ischemia. However, it is important to consider it can be secondary to highly diverse causes, including infection, inflammation, and iatrogenic reasons. The onset of it can occur in patients with inflammatory bowel disease, and when a clinically benign finding, conservative management may be employed [4].

Another case study reports an 18-year-old female patient who presented to the emergency department with intermittent episodes of chronic right abdominal pain, distension, emesis and episodes of nonbloody diarrhea for the past two years which had worsened over the past few days. Contrast abdominal CT was performed and was significant for segmental thickening of the jejunum and ileum walls, mesenteric adenitis, and pneumatosis intestinalis of the jejunum. Magnetic resonance enterography (EnteroMRI) of the abdomen revealed findings similar to those described in the CT. Histological studies revealed flattening of the villi, abundant lymphocyte-dominated infiltration of the lamina propria with polymorphonuclear cells permeating the glandular epithelium. Fecal calprotectin was elevated. Given the clinical, imaging, endoscopic and histological findings, the diagnosis of Crohn’s disease was confirmed. The patient was started on systemic steroids and immunomodulatory management. The authors suggest that in this patient, the chronic inflammatory process of the mucosa associated with Crohn’s disease contributed to the findings of pneumatosis intestinalis appearing on imaging [5].

Finally, a systematic review was conducted to record the cases of concomitant pneumatosis intestinalis and inflammatory bowel disease. The investigators identified 11 cases of pneumatosis intestinalis of which four patients had ulcerative colitis, six had Crohn’s disease and one had indeterminate colitis [6]. Another study reports the prevalence of pneumatosis intestinalis in Crohn’s disease, and reports a prevalence of up to 12% seen on CT enteroscopy [6].

## Conclusions

Pneumatosis intestinalis is an abnormal occurrence and may correlate with underlying pathology. In the setting of the appropriate clinical presentation, diagnostic imaging findings, endoscopic and histologic evidence, inflammatory bowel disease such as Crohn’s disease should be considered as the culprit of pneumatosis intestinalis. This is notably depicted in our patient as PI was observed prior to the double balloon enteroscopy ruling out that it was a consequence of the procedure. Oftentimes, patients may be asymptomatic and should be managed with medical conservative treatment. Additionally, the underlying Crohn’s disease should be managed with appropriate treatment.

## References

[1] Ranasinghe IR, Hsu R (2023). Crohn disease. In: StatPearls [Internet].

[2] Im J, Anjum F (2023). Pneumatosis intestinalis. In: StatPearls [Internet].

[3] Albayrak Y, Aslan S, Kurt A, Bayraktutan ÜG (2011). Pneumatosis intestinalis developing in association with Crohn’s disease and mimicking gastrointestinal system perforation. Iran Red Crescent Med J.

[4] González-Olivares C, Palomera-Rico A, Blanca NRP, Sánchez-Aldehuelo R, Figueroa-Tubío A, García de la Filia I, López-Sanromán A (2019). Pneumatosis intestinalis in Crohn's disease. Gastroenterol Hepatol.

[5] Vargas Rubio R, Bejarano Rengifo J, Ardila Silva E (2020). Pneumatosis intestinalis as a presentation of Crohnâ€™s disease: a case report. Rev Gastroenterol Peru.

[6] Gao Y, Uffenheimer M, Ashamallah M, Grimaldi G, Swaminath A, Sultan K (2020). Presentation and outcomes among inflammatory bowel disease patients with concurrent pneumatosis intestinalis: a case series and systematic review. Intest Res.

